# Damage Characteristics of a Step Lap Joint Exposed to Flexural Loading for Its Different Configurations

**DOI:** 10.3390/polym15112458

**Published:** 2023-05-25

**Authors:** Murat Demiral, Ferhat Kadioglu

**Affiliations:** 1 College of Engineering and Technology, American University of the Middle East, Egaila 54200, Kuwait; 2 Department of Aerospace Engineering, Ankara Yildirim Beyazit University, Ankara 06010, Turkey; fkadioglu@ybu.edu.tr; 3 Department of Aerospace Engineering and Engineering Mechanics, The University of Texas at Austin, Austin, TX 78712, USA

**Keywords:** step lap joints, four-point bending, cohesive zone model, energy absorption, step number

## Abstract

Step lap joints are kinds of lap structures, where butted laminations of each layer are consecutively offset in succeeding layers in the same direction. They are mainly designed this way to reduce the peel stresses at the edges of the overlap area observed in single lap joints. In their service, lap joints are often subjected to bending loads. However, the performance of a step lap joint under flexural loading has not been studied in the literature yet. For this purpose, 3D advanced finite-element (FE) models of the step lap joints were developed via ABAQUS-Standard. DP 460 and A2024-T3 aluminum alloy were used for the adhesive layer and adherends, respectively. The polymeric adhesive layer was modelled using cohesive zone elements with quadratic nominal stress criteria and power law interaction of the energies to characterize the damage initiation and damage evolution, respectively. A surface-to-surface contact method with a penalty algorithm and a hard contact model was used to characterize the contact between the adherends and the punch. Experimental data were used to validate the numerical model. The effects of the configuration of the step lap joint on its performance in terms of the maximum bending load and the amount of energy absorbed were analyzed in detail. A step lap joint with three steps (three-stepped lap joint) was found to show the best flexural performance, and when the overlap length at the upper and lower steps was increased, the amount of energy absorbed by the joint increased markedly.

## 1. Introduction

Lap joints have been increasingly used in advanced engineering applications including the aerospace, construction, automobile, military and marine industries, among others [1]. Their main advantage over traditional methods such as welded, riveted or bolted joints is the reduction in stress concentrations, leading to an enhanced fatigue life, damage tolerance, lightness, ease of manufacturing, etc. [2].

Step lap joints (StLJ), a type of lap joint, are designed to increase the strength of the joints by reducing the peeling stresses developing at the edges of the overlap area mostly observed in single lap joints. In a step lap joint, butted laminations of each layer are offset in the same direction on consecutive levels. 

In the literature, a few studies are available related to step lap joints. For instance, Kimiaeifar et al. [3] assessed the reliability and probability of failure for these joints subjected to bending loads. Kim et al. [4] studied their fatigue characteristics for composite structures under tensile loads. Cracks were observed to initiate at the end of the overlap length and spread through the delamination of the composite adherend. Li et al. [5] proposed a semi-analytical model accounting for transverse shear deformations and rotation of the laminated plates with a stepped lap repair. Ichikawa et al. [6] performed a three-dimensional finite element (FE) analysis for stepped lap adhesive joints subjected to static tensile loading. It was observed that the maximum principal stress occurred at the edge of the adhesive interface and that value decreased with an increase in the step number and a decrease in the adhesive thickness. Sawa et al. [7] developed a three-dimensional FE model alongside experiments to explore the interface stress distribution in stepped lap joints with similar and dissimilar adherends under a bending moment. It was reported that a joint with identical adherends was stronger than that with different ones. Akpinar et al. [8] experimentally and numerically evaluated the strength of step lap joints with different numbers of steps and different adhesives subjected to tensile loading. It was observed that three- and single-step lap joints increased the load carrying capacity by more than 8.0% and 60.0%, respectively, when compared to single lap joints. In another study, Durmus and Akpinar [9] investigated the influence of step length on the tensile performance of the joint. It was reported that when the step lengths at different heights were close to each other, the failure load of the joint increased substantially. Mistry et al. [10] performed a similar FE study, but for thicker adherends and joints which were subjected to both tensile and bending loadings. A three-stepped lap joint with 2-21-2 mm configuration (2 mm overlap lengths at the top and bottom and 21 mm at the middle height) was found to be the most suitable in terms of the level of stress and deformation for both loadings. Gavgali et al. [11] performed an investigation on the fatigue performance of single lap and step lap joints subjected to tensile and bending loadings experimentally and numerically. This study revealed that applying three-step lap joints on the overlap area decreased the fatigue strength limit of the joint significantly under bending loading when compared to a single lap joint. 

Lap joints are often subjected to bending loads [12,13]. For instance, in the aviation sector, the weight of the aircraft, the forces produced by its engines and the aerodynamic forces acting on its wings and other surfaces all contribute to the generation of bending forces. These loads can cause stress and deformation in the aircraft’s components such as the wings, the fuselage and the joints. Engineers must take into account the bending loads that an airplane might experience when constructing structures so that they possess the rigidity and structural strength required to withstand these loads. However, it can be seen from the above studies that most of the research performed on step lap joints is related to tensile loading conditions. Additionally, in studies [3,10,11], the flexural failure response of the stepped lap joints was not explained in detail. For this purpose, in this study, an advanced three-dimensional FE model of a step lap joint subjected to a four-point bending load using Abaqus/Standard was developed. The adhesive layer was modelled using cohesive zone modelling with a traction–separation response, where the quadratic nominal stress criteria and power law interaction of the energies were used to characterize the damage initiation and evolution, respectively. The contact between the adherends and the punch was modelled using the surface-to-surface contact method with the penalty algorithm and the hard contact model. The goal of this paper is to elucidate the complex failure process of the adhesive layer in stepped lap joints in different configurations under bending loading [14,15,16].

The paper is structured as follows. Section 2 presents the numerical model developed with a brief description of the cohesive zone modelling. In Section 3, the verification of the developed FE model with experiments from the literature is shown, followed by the results and discussion of the influence of the lengths and number of steps in the joint on the failure characteristics of the adhesive layer. The conclusion is presented in Section 4. 

## 2. Numerical Modelling

### Finite Element Modelling

**Figure 1:**
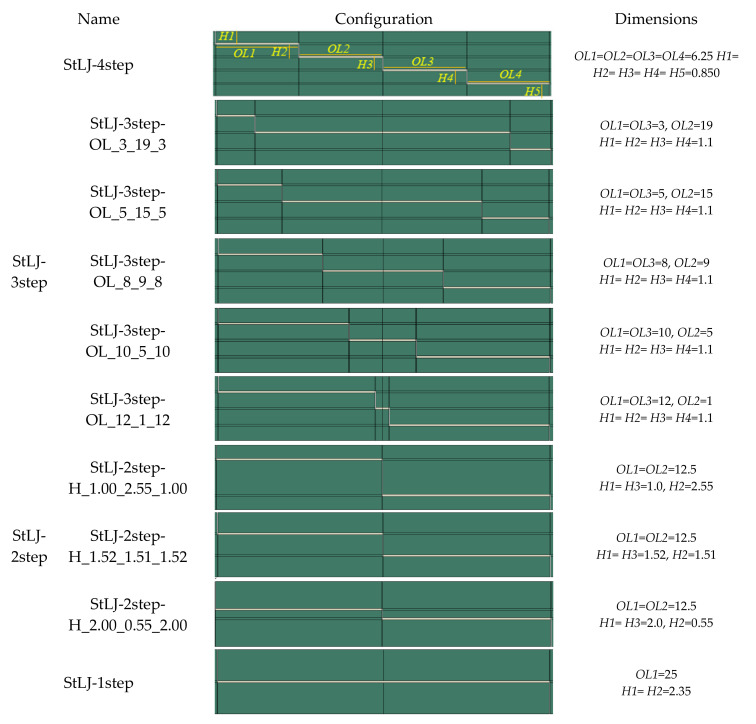
Geometric depictions and labels of different StLJ configurations.

Adhesively bonded stepped lap joints with different configurations under four-point bending were analyzed. Their geometric details with their self-descriptive labels are given in Figure 1. Firstly, the number of steps was changed from 1 to 4 and they are labelled as StLJ-1step, StLJ-2step, StLJ-3step and StLJ-4step, respectively. Secondly, keeping the step number as 3, the overlap length (*OL*) at different steps was varied. For instance, this length at the highest step, identical to that at the lowest step, was increased from 3.0 mm (StLJ-3step-OL_3_19_3) to 12.0 mm (StLJ-3step-OL_12_1_12) successively. Thirdly, keeping the step number at 2, the relative height (*H*) of different steps was changed keeping those of the highest (*H*1) and lowest steps identical (*H*3). For instance, *H*1 was changed from 1.52 mm to 1.0 mm and 2.0 mm. It should be mentioned that, unless otherwise stated, StLJ-2step refers to StLJ-2step-H_1.52_1.51_1.52 and StLJ-3step refers to StLJ-3step-OL_8_9_8. 

**Figure 2:**
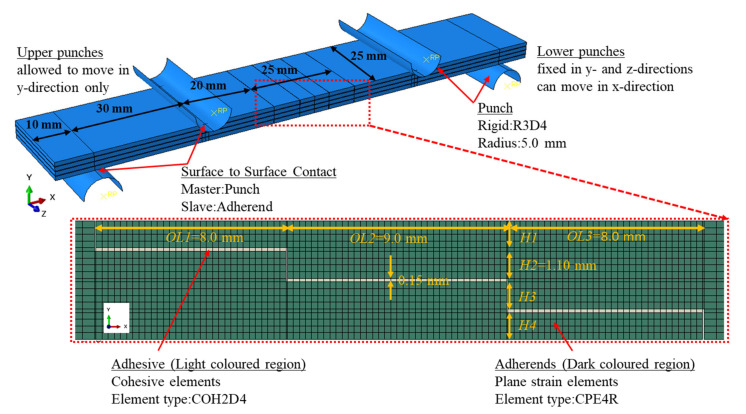
Details of the three-dimensional FE model of adhesively bonded three stepped-lap joint (StLJ-3step-OL_8_9_8) under four point bending.

The three-dimensional developed FE model of a three-stepped lap joint under four-point bending is shown in Figure 2. The model was created using ABAQUS/Standard. Different configurations for the overlap region were considered as described in Figure 1. The dimensions of the model were chosen in accordance with those in [11], where the experimental data in this reference were used to validate the FE model. The adherends are 85 mm, 4.85 mm and 25 mm in length, height and width, respectively, while the thickness of the adhesive layer is 0.15 mm. 

The behaviors of adherends and adhesive layer were modeled using eight-node three-dimensional cohesive (COH3D8) and eight-node linear brick reduced integration (C3D8R) elements, respectively. Outside of the overlap region, a coarse mesh with an average element size of 1.0 mm in all directions was used, except for the region where the punch contacts the adherends, at this point, 0.25 mm was used. On the other hand, a finer mesh was used in the overlap region. A mesh convergence study was performed in this region to characterize the failure of the adhesive layer properly. Element sizes of 0.5 mm × 0.5 mm × 0.5 mm (coarsest), 0.25 mm × 0.25 mm × 0.25 mm (medium) and 0.125 mm × 0.125 mm × 0.125 mm (finest) were considered. Table 1 demonstrates the maximum bending loads (*F_b,max_*) obtained using different mesh sizes. As the difference between the bending loads obtained with the medium and finest meshes is less than 5.0%, an element size of 0.25 mm × 0.25 mm × 0.25 mm was selected for the simulations. That enabled us to reduce the computational time significantly from an average of 38 h (finest) to 4.75 h (medium) using 12 Intel quad-core processors. On the other hand, the punches with a radius of 5.0 mm were modelled as rigid bodies with an element type of R3D4 and element size of 0.5 mm.

Surface-to-surface contact was defined between the punch and the adherend material, where the former was the master and the latter was the slave. A penalty algorithm with a friction coefficient of 0.3 and a hard contact model characterized the contact between the bodies. The punches under the adherends were fixed in the *y-* and *z-*directions, but allowed to move in *x-*direction, while the ones above the adherends were allowed to move in the *y*-direction only. 

Standard elasto-plastic constitutive equations were used to analyze the adherents’ behavior. The behavior of the adhesive layer was simulated using the cohesive zone modelling scheme. In the following, a brief description is given.

**Figure 3:**
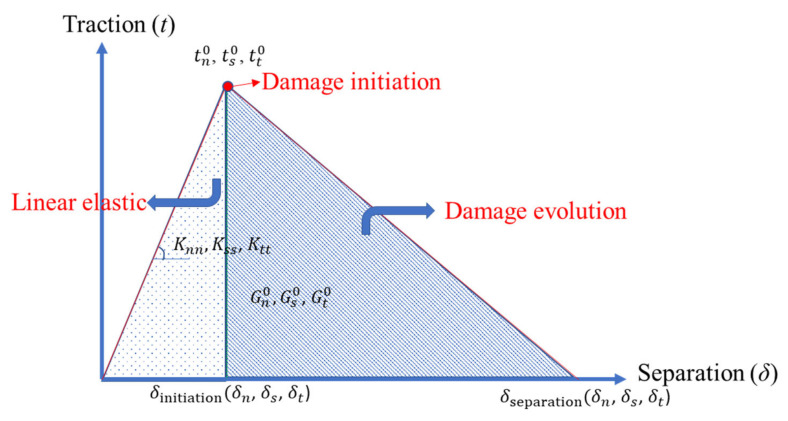
The bilinear traction separation law with the parameters used in the cohesive zone model.

Following the studies [11,17], the bilinear traction separation law governed the behavior of the cohesive element, where its parameters are presented in Figure 3. The initial loading part follows linear elastic behavior with the following equation:

(1) $$t=\left[\begin{array}{c} t_{n}\\ t_{s}\\ t_{t} \end{array}\right]=\left[\begin{array}{ccc} K_{nn} & 0 & 0\\ 0 & K_{ss} & 0\\ 0 & 0 & K_{tt} \end{array}\right]\left[\begin{array}{c} \delta_{n}\\ \delta_{s}\\ \delta_{t} \end{array}\right]$$

where $t_{n}$, $t_{s}$ and $t_{t}$ represent the tractions in normal (Mode 1), shear (Mode 2) and tangential (Mode 3) directions, respectively, and $\delta_{i}$ and $K_{ii}$ (*i* = *n*, *s*, *t*) are the separation and initial stiffness, respectively, of the cohesive element in each direction.

The mechanism of failure has two parts: damage initiation and damage evolution. The initiation of damage is characterized using a quadratic traction function as the summation of nominal stress ratios reaching one, as in the following:

(2) $${\left\{\frac{\langle t_{n}\rangle }{t_{n}^{0}}\right\}}^{2}+{\left\{\frac{t_{s}}{t_{s}^{0}}\right\}}^{2}+{\left\{\frac{t_{t}}{t_{t}^{0}}\right\}}^{2}=1$$

where 〈.〉. is the Macaulay bracket, $t_{n}^{0}$, $t_{s}^{0}$ and $t_{t}^{0}$ are the strength of the adhesive layer for modes 1 to 3, respectively, and $t_{n}$, $t_{s}$ and $t_{t}$ are the resulting stresses in the normal, shear and tangential directions, respectively. The power law fracture criterion is used for damage evolution in the adhesive layer as follows:

(3) $${\left\{\frac{\langle G_{n}\rangle }{G_{n}^{0}}\right\}}^{n}+{\left\{\frac{G_{s}}{G_{s}^{0}}\right\}}^{n}+{\left\{\frac{G_{t}}{G_{t}^{0}}\right\}}^{n}=1$$

In this expression, $G_{n}^{0}$, $G_{s}^{0}$ and $G_{t}^{0}$ represent the critical fracture energies for the above-mentioned failure modes, $G_{n}$, $G_{n}$ and $G_{n}$ denote the energies by the pure mode tractions ($t_{i}$) and their conjugate separations ($\delta_{i}$) in the normal, first and second shear directions, respectively, and *n* is the power law constant.

In the damage evolution region, the constitutive equations for mixed-mode loading with the single damage variable *SDEG* describing the material loss of stiffness for all the modes are as follows:

(4) $$t_{n}=K_{nn}\left(1-SDEG\right)\langle \delta_{n}\rangle -K_{nn}\langle -\delta_{n}\rangle$$

$$t_{s}=K_{ss}\left(1-SDEG\right)\delta_{s}$$

$$t_{t}=K_{tt}\left(1-SDEG\right)\delta_{t}$$

In the analysis, the *SDEG* is calculated using an equivalent traction–separation curve, where the latter is formulated as follows:

(5) $$\delta_{equivalent}=\sqrt{\langle \delta_{n}\rangle {}^{2}+\delta_{s}{}^{2}+\delta_{t}{}^{2}}$$

Then, the cosine directions ($B_{n}$, $B_{s}$ and $B_{t}$) of the $\delta_{equivalent}$ are defined as:

(6) $$B_{n}=\cos\beta_{n}=\frac{\langle \delta_{n}\rangle }{\delta_{equivalent}}\;B_{s}=\cos\beta_{s}=\frac{\delta_{s}}{\delta_{equivalent}}\;B_{t}=\cos\beta_{t}=\frac{\delta_{t}}{\delta_{equivalent}}$$

where $\beta_{n}$, $\beta_{s}$ and $\beta_{t}$ are the angles between the coordinate axes of the related modes and the equivalent displacement vector.

The equivalent specific energy can be calculated as the sum of the specific energies for different modes, $G_{equivalent}$ = $G_{n}\;$+ $G_{s}+G_{t}$, where the individual terms are expressed as [18]:

(7) $$G_{n}=\left\{\begin{array}{cc} \frac{1}{2}K_{nn}B_{n}^{2}\delta_{equivalent\;}^{2} & \;if\;\delta_{equivalent\;}\leq \delta_{initiation\;}\\ \frac{1}{2}K_{nn}B_{n}^{2}\delta_{initiation\;}\left(\delta_{separation\;}-\frac{{\left(\delta_{separation\;}-\delta_{equivalent\;}\right)}^{2}}{\left(\delta_{separation\;}-\delta_{initiation\;}\right)}\right) & \;if\;\delta_{initiation\;}\leq \delta_{equivalent\;}\leq \delta_{separation\;}\\ \frac{1}{2}K_{nn}B_{n}^{2}\delta_{equivalent\;}\delta_{separation\;} & \;if\;\delta_{equivalent\;}\geq \delta_{separation\;} \end{array}\right.\;G_{s}=\left\{\begin{array}{cc} \frac{1}{2}K_{ss}B_{s}^{2}\delta_{equivalent\;}^{2} & \;if\;\delta_{equivalent\;}\leq \delta_{initiation\;}\\ \frac{1}{2}K_{ss}B_{s}^{2}\delta_{initiation\;}\left(\delta_{separation\;}-\frac{{\left(\delta_{separation\;}-\delta_{equivalent\;}\right)}^{2}}{\left(\delta_{separation\;}-\delta_{initiation\;}\right)}\right) & \;if\;\delta_{initiation\;}\leq \delta_{equivalent\;}\leq \delta_{separation\;}\\ \frac{1}{2}K_{ss}B_{s}^{2}\delta_{equivalent\;}\delta_{separation\;} & \;if\;\delta_{equivalent\;}\geq \delta_{separation\;} \end{array}\right.\;G_{t}=\left\{\begin{array}{cc} \frac{1}{2}K_{tt}B_{t}^{2}\delta_{equivalent\;}^{2} & \;if\;\delta_{equivalent\;}\leq \delta_{initiation\;}\\ \frac{1}{2}K_{tt}B_{t}^{2}\delta_{initiation\;}\left(\delta_{separation\;}-\frac{{\left(\delta_{separation\;}-\delta_{equivalent\;}\right)}^{2}}{\left(\delta_{separation\;}-\delta_{initiation\;}\right)}\right) & \;if\;\delta_{initiation\;}\leq \delta_{equivalent\;}\leq \delta_{separation\;}\\ \frac{1}{2}K_{tt}B_{t}^{2}\delta_{equivalent\;}\delta_{separation\;} & \;if\;\delta_{equivalent\;}\geq \delta_{separation\;} \end{array}\right.$$

The equivalent damage onset displacement ($\delta_{initiation}$) and citical displacement ($\delta_{separation}$) for mixed-mode loading can be obtained as follows:

(8) $$\delta_{initiation}{}^{2}={\left[{\left(\frac{K_{nn}B_{n}}{t_{n}^{0}}\right)}^{2}+{\left(\frac{K_{ss}B_{s}}{t_{s}^{0}}\right)}^{2}+{\left(\frac{K_{tt}B_{t}}{t_{t}^{0}}\right)}^{2}\right]}^{-1}\;\delta_{separation}=\frac{2}{\delta_{initiation}K}{\left[\frac{\emptyset_{n}}{G_{n}^{0}}+\frac{\emptyset_{s}}{G_{s}^{0}}+\frac{\emptyset_{t}}{G_{t}^{0}}\right]}^{-1}$$

Here, the equivalent stiffness K equals $K_{nn}B_{n}{}^{2}+K_{ss}B_{s}{}^{2}+K_{tt}B_{t}{}^{2}$ and the mode ratios $\emptyset_{n}$, $\emptyset_{s}$ and $\emptyset_{t}$ are $G_{n}/G_{equivalent}$, $G_{s}/G_{equivalent}$ and $G_{t}/G_{equivalent}$, respectively. Then, *SDEG* can be calculated as follows:

(9) $$SDEG=\frac{\delta_{separation}\left(\delta_{equivalent}-\delta_{initiation}\right)}{\delta_{equivalent}\left(\delta_{separation}-\delta_{initiation}\right)}$$

The damage evolution on the adhesive layer was monitored using the parameter *SDEG* in the simulations. Its value ranges between 0.0 and 1.0, meaning no damage and complete damage at the integration point in the cohesive elements, respectively [17]. When *SDEG* equals 1.0 at all integration points of an element, this element cannot carry force anymore and hence it is deleted from the model. The material parameters of the adherends and adhesive layer employed in the simulations are shown in Table 2. 

## 3. Results and Discussion

In this section, firstly, the numerical model was validated using the experimental data taken from the literature. The influence of different parameters defining the configurations of the StLJ on the bending performance of the joint was then thoroughly studied.

### 3.1. Validation of the FE Model

**Figure 4:**
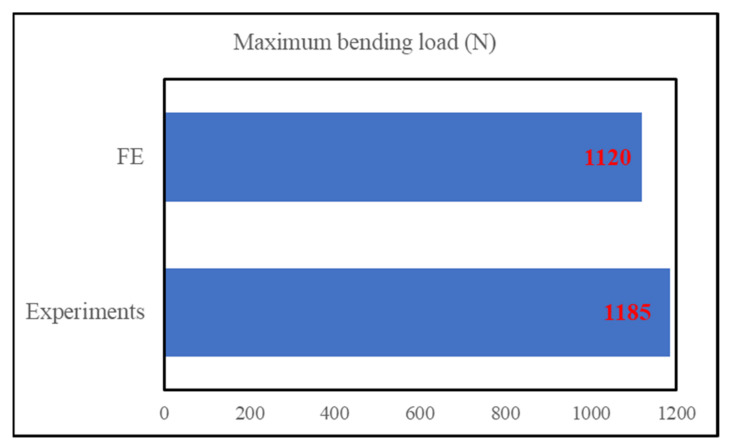
Comparison of maximum bending load obtained from experiment [11] and FE simulations.

The developed FE model for three-stepped lap joint was validated for the bending load with the one obtained experimentally in [11]. The FE simulations demonstrated that the bending loads measured from the left and right punches were different, as will be shown in the next part. As the failure of the adhesive layer started from the right end, the punch on that side reached its maximum value earlier, while the force on the other punch kept increasing. Therefore, the overall maximum force was measured from the left punch mostly and this value is used here. Figure 4 compares the experimentally and numerically obtained maximum bending loads. They are 1185 N and 1120 N. As the difference between them is around 5.0%, the numerical model is considered to be validated. 

### 3.2. Effect of Number of Steps

**Figure 5:**
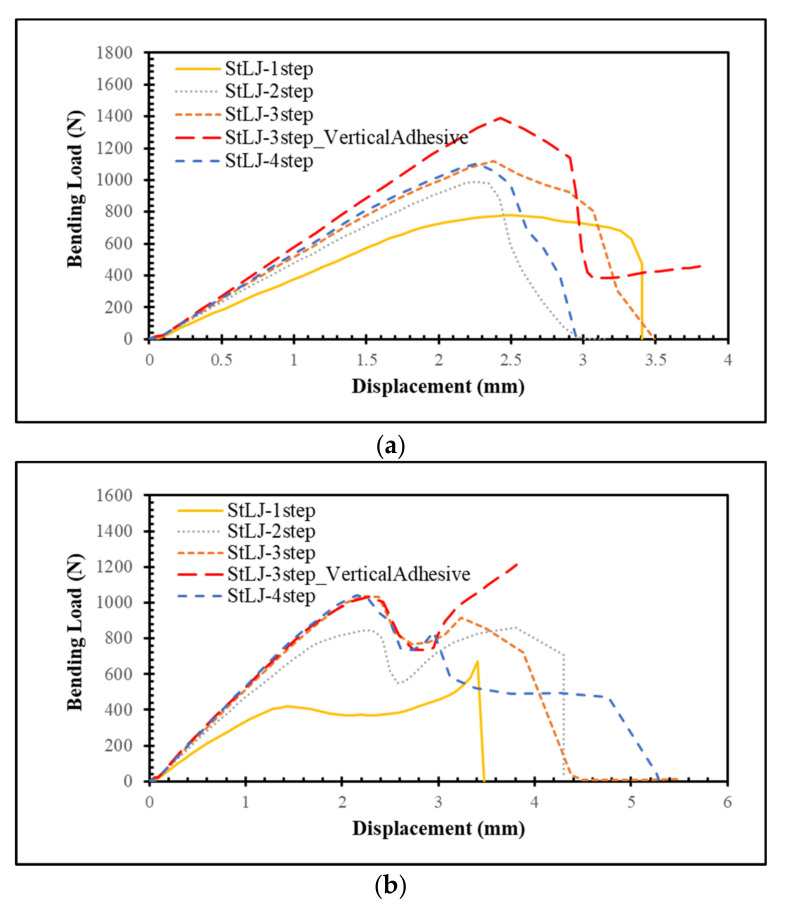
Force–displacement behavior of StLJs with different numbers of steps obtained via readings from the left (**a**) and right (**b**) punches.

**Figure 6:**
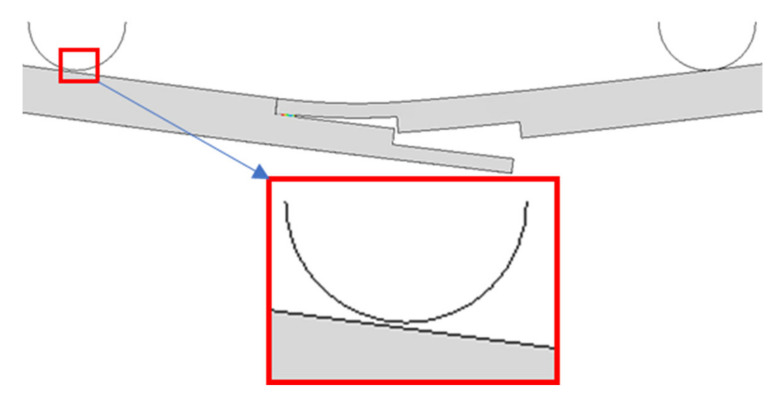
The contact position between the left punch and the adherends during the failure of two-stepped lap joint.

This section investigated how the number of steps in the lap joint affected the StLJs bending load response. To this end, one to four stepped joint configurations were compared and contrasted. Figure 5 compares their bending load displacement curves obtained from left and right punches separately. It can be observed that the maximum loads the StLJ carries is 782 N, 986 N, 1120 N and 1102 N, respectively, for the left punch, while they were 420 N, 844 N, 1034 N and 1040 N, respectively, for the right punch. It was noted that the force applied by the left side punch decreased after reaching its maximum, while a similar one was also noticed for the other punch, but with a key difference being that a second peak was observed for the latter. This was due to the fact that when the StLJ failed, the contact between the left punch and the adherend was lost and only the right punch continued to apply deformation (see Figure 6). This could be also observed from Figure 5. For instance, when the load on the left punch started to decrease for StLJ-2step at 2.4 mm, a similar behavior was also observed for the right punch, but then the force on this punch started to rise again at 2.6 mm, while the force on left punch kept decreasing and reached zero, meaning no contact with the sample. Overall, it was noticed that with an increase in the number of steps, the maximum load that the StLJ carried increased, where the three- and four-stepped configurations had very similar highest loads. 

To measure the capacity of a step lap joint absorbing energy subjected to flexural loading, the total energy absorption (*EA*) is calculated. It is defined as the integration of the force (*F*) versus displacement (*δ*) curve, i.e., the area under the force–displacement curve [19], as follows:

(10) $$EA=\overset{\delta }{\underset{0}{\int }}F\left(\delta \right)d\delta$$

Table 3 presents the *EA* obtained from Figure 5 for different StLJs calculated for two punches. It is seen that 2.949 J, 3.461 J, 4.867 J and 3.831 J are the total energies absorbed for one- to four-stepped joints, respectively. The three-step lap joint absorbed the highest amount of energy among them. In fact, when the peak load was achieved for StLJ-3step, it did not reach zero when the punch travelled shorter distances, as in the case of StLJ-4step; therefore, more energy could be absorbed. On the other hand, StLJ-1step absorbed more energy than StLJ-2step considering only the left punch, with the values of 1.779 J and 1.170 J, respectively. However, the opposite behavior was noted for the right-hand-side punch. Overall, the latter absorbed more energy. Here, we can conclude that StLJ-3step is the best configuration in terms of carrying the highest bending load and amount of energy absorbed. 

**Figure 7:**
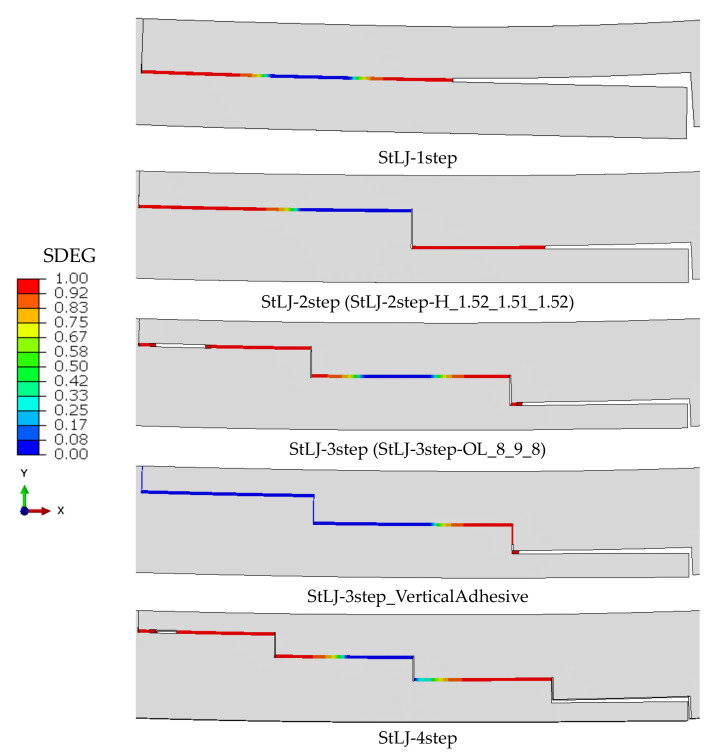
Distribution of damage (*SDEG*) on the adhesive layer upon maximum load was attained from the left side punch.

Figure 7 presents the damage distribution, i.e., *SDEG*, on the adhesive layer when the maximum load is attained on the left punch. For all of them, the damage started from the right end of the adhesive layer and later from the left side, then from both sides, propagating towards to the center [20]. An interesting point here is that for StLJ-1step and StLJ-3step, the crack propagated more from the right side towards to the center of the adhesive layer when compared to the other two configurations. This was due to the fact that as the whole or partial overlap length was at the neutral axis for the first two step lap joints, they could not carry the load as efficiently as the others, and therefore the crack penetrated more. 

In the design of StLJs in this study, the adhesive was used only along the horizontal faces of the steps of the adherends, whereas no adhesive was used along their vertical faces. An additional simulation was also performed to see the importance of joining two adherends along their vertical edges for StLJ-3step. It is labelled as StLJ-3step_VerticalAdhesive. Figure 5 and Figure 7 as well as Table 3 presents the results. Due to convergency issues, the simulation was not completed; therefore, in the *F(δ)* curves, the force did not reach zero. It was observed that *F_b,max_* from the left punch increased from 1120 N to 1391 N, while the values obtained from the right punch revealed that first part of the *F(δ)* curve was very similar, but a larger force was achieved for the second peak, even larger than the first peak. From the *SDEG* distribution in Figure 7, it is observed that the damage distribution of the adhesive layer changed significantly when the crack initiated only from the right bottom side and the vertical adhesive used prevented the initiation of damage on the top left side. Therefore, a much higher flexural strength was achieved. That can be seen also from its *EA* value. This value increased by 13.33% from 4.867 J to 5.516 J, even without adding the energy values in the final parts of *F–(δ)* curves, as the simulation was not completed. 

### 3.3. Influence of Overlap Lengths at Various Steps

**Figure 8:**
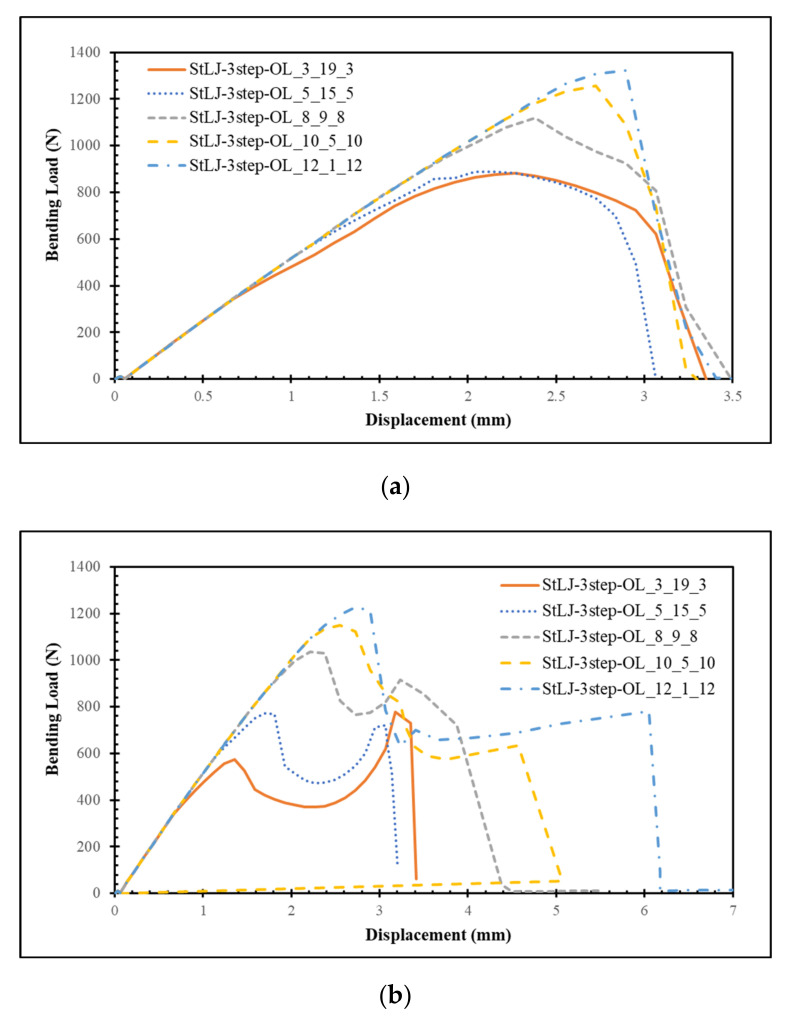
Force–displacement behavior of StLJ-3step with different overlap lengths at different steps obtained via readings from the left (**a**) and right (**b**) punches.

In this part, the effects of partial distribution of the overlap length at different steps of StLJ-3step on its performance were investigated. Here, the overlap length at the mid-height (intermediate step) of the StLJ-3step was changed from 19 mm (StLJ-3step-OL_3_19_3) to 1 mm (StLJ-3step-OL_12_1_12) gradually while keeping the outer ones identical. Figure 8 shows the F–δ curves obtained from different punches. It was noticed that the maximum force was attained when StLJ-3step-OL_12_1_12 was used, with values of 1322 N and 1226 N from the readings of the left and right punches, respectively. The characteristics of the curves are the same as explained in Section 3.2. Overall, with a decrease in the overlap length at the middle step, the maximum force increased. Table 4 shows the EA values for all configurations. Likewise, more energy was absorbed when the mid-height OL decreased. Interestingly, the amount of energy increased more than 100% from 3.300 J to 6.762 J when this OL changed from 19 mm to 1 mm. These two observations from F–δ and energy absorption could be explained by the fact that as the adhesive layers close to the top side or bottom side of the joint are exposed to a larger bending load compared to those lying at the neutral axis, the StLJs with a larger portion of OLs lying at the top or bottom sides rather than the middle are subjected ti larger bending forces and also absorb more energy. 

### 3.4. Influence of the Relative Heights of Different Steps 

**Figure 9:**
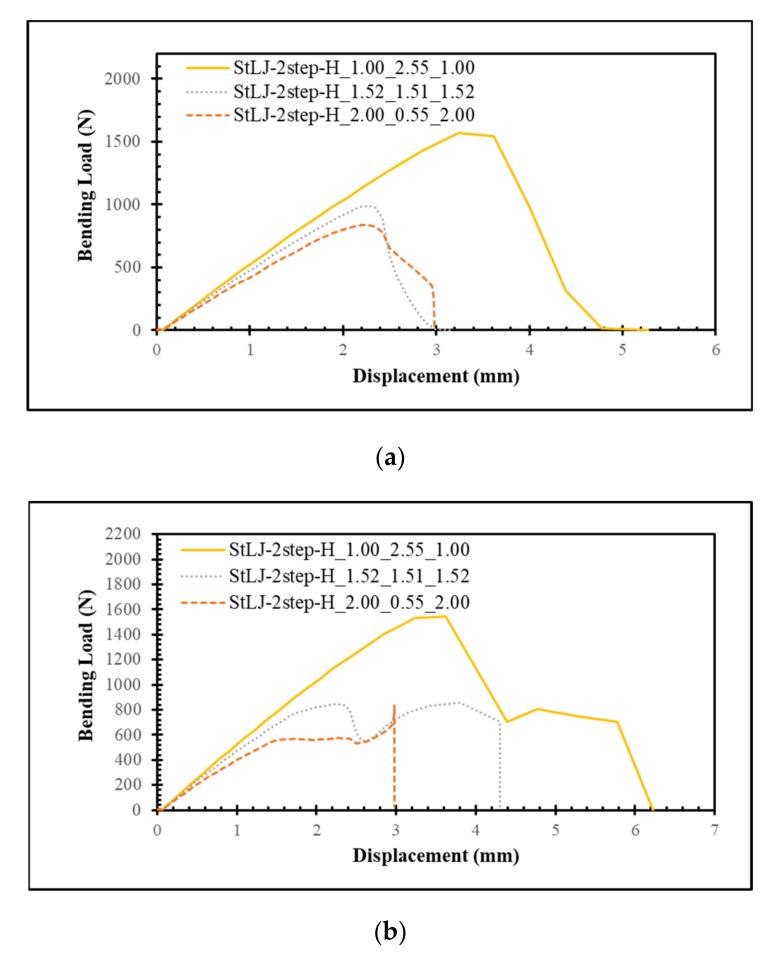
Force–displacement behavior of StLJ-2step with different heights of the steps obtained via readings from the left (**a**) and right (**b**) punches.

In this last section, the relative height positions of different steps were varied to evaluate its effect on the performance of the StLJ. For this purpose, two-stepped lap joints with three different heights of both the upper and lower steps were considered (see Figure 1), they are namely 1.00 mm, 1.52 mm and 2.00 mm. Their *F*(*δ)* curves and *EA* values are presented in Figure 8 and Table 5, respectively. The *F_b,max_* from the left punch is 1571 N, 986 N and 838 N, respectively (see Figure 9). When the corresponding *EA* values are compared using Table 5, it is seen that the amount of energy absorbed decreased dramatically from 9.366 J to 4.176 J and later to 2.758 J when the heights of both upper and lower steps were increased from 1.00 mm into 1.52 mm first and later to 2.00 mm. Here, it is concluded that when the heights of both upper and lower steps are decreased, these parts of the StLJ become further from the neutral axis. Therefore, they start to carry higher bending loads and absorb more energy. 

## 4. Concluding Remarks

This work studied the bending performance of a step lap joint with different configurations subjected to four-point bending. For this purpose, 3D FE models were developed, where the behavior of the adhesive layer was modelled using cohesive zone elements. The model was first validated using experimental data from the literature, then the effects of different parameters of the step lap joint were investigated. 

The key findings are as follows:

- The three-stepped lap joint was found to carry the highest bending load and absorbed the highest amount of energy when compared to one-, two- and four-stepped lap joints.
- It is suggested to join the adherends using adhesives not only along their horizontal faces, but also along their vertical face for StLJs. By doing so, reasonable increases in *F_bmax_* and *EA* can be attained.
- With an increase in the overlap length at the upper and lower steps, i.e., with the smallest portion at the mid step, the joint showed the best flexural performance. In this configuration, a more than 100% increase in EA was achieved.
- In line with the above point, when the upper and lower steps of the step lap joint were placed closer to the top and bottom surfaces of the joint, an around 1.87-fold increase in the *F_bmax_* and an aroundp 3.40-fold increase in the EA were achieved.
- In stepped lap joints subjected to bending loads, portions of the adhesive layer located at or close to the neutral axis do not markedly contribute to the flexural strength.

## Figures and Tables

**Table 1:** Mesh analysis based on the maximum bending load.

Mesh Size	*F_b,max_* (N)
0.5 mm × 0.5 mm × 0.5 mm	1230
0.25 mm × 0.25 mm × 0.25 mm	1120
0.125 mm × 0.125 mm × 0.125 mm	1109

**Table 2:** The material constants for AA2024-T3 and DP460 used in FE simulations [11].

AA2024-T3	*E* (MPa)	ϑ	$\sigma_{Y}$ (MPa)			
	72,400	0.33	324			
DP460	$K_{nn}\;K_{ss}\;K_{tt}$ (N/mm^3^)	$t_{n}^{0}$ (MPa)	$t_{s}^{0}$ $t_{t}^{0}$ (MPa)	$G_{n}^{0}$ (N/mm)	$G_{s}^{0}\;G_{t}^{0}$ (N/mm)	*n*
	10^5^	32.6	28.5	2.56	11.71	2.0

**Table 3:** Comparison of energy absorption performances of StLJs with different number of steps obtained from different punches and their summation.

	*EA* (J)		
Configuration	Left Punch	Right Punch	Sum
StLJ-1step	1.779	1.170	2.949
StLJ-2step	1.515	1.946	3.461
StLJ-3step	2.227	2.640	4.867
StLJ3step_VerticalAdhesive	2.742	2.774	5.516
StLJ-4step	1.819	2.012	3.831

**Table 4:** Comparison of energy absorption performances of StLJ-3step with different overlap lengths at different steps obtained from different punches and their summation.

	*EA* (J)		
Configuration	Left Punch	Right Punch	Sum
StLJ-3step-OL_3_19_3	1.890	1.410	3.300
StLJ-3step-OL_5_15_5	1.774	1.555	3.329
StLJ-3step-OL_8_9_8	2.227	2.834	5.061
StLJ-3step-OL_10_5_10	2.286	3.275	5.561
StLJ-3step-OL_12_1_12	2.370	4.392	6.762

**Table 5:** Comparison of energy absorption performances of StLJ-2step with different heights of the steps obtained from different punches and their summation.

	*EA* (J)		
Configuration	Left Punch	Right Punch	Sum
StLJ-2step-H_1.00_2.55_1.00	4.067	5.299	9.366
StLJ-2step-H_1.52_1.51_1.52	1.515	2.661	4.176
StLJ-2step-H_2.00_0.55_2.00	1.469	1.289	2.758

## Data Availability

The data presented in this study are available on request from the corresponding author.
